# Supplementary material for: Blood Levels of Endocannabinoids, Oxylipins, and Metabolites Are Altered in Hemodialysis Patients
Source: Int J Mol Sci. 2022 Aug 29;23(17):9781. doi: 10.3390/ijms23179781 (PMC9456435; doi:10.3390/ijms23179781)
Supplement: Supplementary file 1 [file ijms-23-09781-s001.zip › New Supplemental Materials Hemo Table S1.pdf]

Supplemental Materials Table S1. Plasma endocannabinoids and oxylipins measured in healthy controls and hemodialysis patients.

Compound	Parent Fatty Acid	Units	Control	HDP	VIP	p-value
			Monoacylglycerols			
1-OG	OA	nM	54.3 ± 34	54.3 ± 24	0.34	0.81
2-OG	""	nM	38.3 ± 13	65.8 ± 38	1.31	0.13
1-LG	LA	nM	6.25 ± 6	10.6 ± 5.9	1.38	0.033
2-LG	""	nM	30.5 ± 15	67 ± 42	1.57	0.026
1-AG	AA	nM	4.4 ± 2.7	4.47 ± 1.8	0.40	0.61
2-AG	""	nM	4.25 ± 1.6	6.45 ± 4.5	0.75	0.30
			N-Acylethanolamides			
P-EA	PA	nM	5.69 ± 1.3	5.78 ± 1.8	0.43	1.00
S-EA	SA	nM	9.44 ± 4.7	13.4 ± 5.8	0.76	0.25
O-EA	OA	nM	14.1 ± 5.1	24.5 ± 9.7	1.82	0.0086
L-EA	LA	nM	5.8 ± 1.5	10.4 ± 4.1	1.91	0.0017
aL-EA	aLEA	nM	0.082 ± 0.03	0.154 ± 0.091	1.46	0.024
DGLA-EA	DGLA	nM	0.432 ± 0.13	0.421 ± 0.1	0.42	0.99
A-EA	AA	nM	1.59 ± 0.3	1.88 ± 0.71	0.95	0.49
D-EA	AdA	nM	0.734 ± 0.19	1.06 ± 0.32	1.60	0.023
DH-EA	DHA	nM	0.374 ± 0.12	0.338 ± 0.15	0.58	0.38
			Prostanoids			
TXB2	AA	nM	0.693 ± 0.81	0.364 ± 0.36	0.65	0.32
6-ketoPGF1a	""	nM	0.177 ± 0.078	0.377 ± 0.34	1.06	0.10
PGE2	""	nM	0.113 ± 0.1	0.126 ± 0.14	0.44	0.95
PGD2	""	nM	0.0938 ± 0.046	0.196 ± 0.2	1.11	0.22
			Triols			
9,12,13-TriHOME	LA	nM	2.51 ± 1.3	1.59 ± 0.6	1.18	0.063
9,10-13-TriHOME	""	nM	3.9 ± 1.9	2.35 ± 0.89	1.45	0.019
			Alcohols			
13-HODE	LA	nM	43 ± 10	45.8 ± 20	0.50	0.95
9-HODE	""	nM	10.7 ± 2.3	10.6 ± 5.3	0.54	0.58
13-HOTE	aLEA	nM	1.04 ± 0.23	1.24 ± 0.47	0.89	0.32
9-HOTE	""	nM	1.32 ± 0.23	1.33 ± 0.5	0.30	0.78
15-HETrE	DGLA	nM	0.43 ± 0.18	0.361 ± 0.2	0.94	0.32
15-HETE	AA	nM	1.66 ± 0.68	1.19 ± 0.51	1.13	0.11
12-HETE	""	nM	15.6 ± 10	9.68 ± 8.4	1.04	0.12
11-HETE	""	nM	0.318 ± 0.13	0.221 ± 0.12	1.05	0.11
9-HETE	""	nM	0.46 ± 0.15	0.316 ± 0.17	1.16	0.070
8-HETE	""	nM	0.532 ± 0.2	0.449 ± 0.16	0.90	0.39
5-HETE	""	nM	1.96 ± 1.6	1.11 ± 0.59	0.94	0.19

12-HEPE	EPA	nM	1.92 ± 1.4	0.76 ± 0.67	1.31	0.04
5-HEPE	""	nM	0.295 ± 0.16	0.147 ± 0.077	1.38	0.03
17-HDoHE	DHA	nM	0.48 ± 0.23	0.33 ± 0.24	0.76	0.31
			Ketones			
13-KODE	LA	nM	7.03 ± 3.7	6.96 ± 4.8	0.69	0.69
9-KODE	""	nM	7.37 ± 4	7.14 ± 4.9	0.60	0.72
5-KETE	AA	nM	0.178 ± 0.083	0.152 ± 0.091	0.81	0.43
			Epoxy-Ketone			
12(13)-Ep-9-KODE	LA	nM	9.77 ± 11	5.05 ± 3.8	0.85	0.19
			Hydroperoxides			
13-HpODE	LA	Fold-C	7.94 ± 7.5	7.68 ± 7.9	0.67	0.40
9-HpODE	""	Fold-C	33.8 ± 34	34 ± 37	0.66	0.38
15-HpETE	AA	Fold-C	18 ± 13	15.2 ± 13	0.74	0.39
12-HpETE	""	Fold-C	142 ± 180	49.1 ± 50	1.09	0.087
5-HpETE	""	Fold-C	2.01 ± 2	0.84 ± 0.66	1.05	0.10
			Epoxides			
12(13)-EpOME	LA	nM	9.98 ± 6.1	8.46 ± 5.3	0.85	0.44
9(10)-EpOME	""	nM	8.24 ± 5.2	6.65 ± 4.1	0.85	0.43
15(16)-EpODE	aLEA	nM	2.01 ± 1.2	1.74 ± 1.2	0.65	0.47
12(13)-EpODE	""	nM	0.367 ± 0.28	0.244 ± 0.14	0.94	0.21
9(10)-EpODE	""	nM	1.09 ± 0.73	1.02 ± 0.66	0.67	0.71
14(15)-EpETrE	AA	nM	0.437 ± 0.28	0.203 ± 0.11	1.51	0.014
11(12)-EpETrE	""	nM	0.551 ± 0.32	0.309 ± 0.17	1.29	0.055
8(9)-EpETrE	""	nM	0.195 ± 0.13	0.0822 ± 0.068	1.02	0.14
17(18)-EpETE	EPA	nM	0.124 ± 0.11	0.0478 ± 0.019	0.96	0.14
19(20)-EpDPE	DHA	nM	0.484 ± 0.38	0.212 ± 0.09	1.44	0.019
			Diols			
12,13-DiHOME	LA	nM	3.64 ± 1.4	3.68 ± 2.8	0.62	0.62
9,10-DiHOME	""	nM	23 ± 8.3	21.1 ± 18	0.70	0.30
15,16-DiHODE	aLEA	nM	6.09 ± 2.6	5.07 ± 2.6	0.83	0.29
9,10-DiHODE	""	nM	0.164 ± 0.084	0.179 ± 0.12	0.23	0.98
14,15-DiHETrE	AA	nM	0.488 ± 0.11	0.488 ± 0.14	0.76	0.92
11,12-DiHETrE	""	nM	0.372 ± 0.084	0.287 ± 0.089	1.34	0.031
8,9-DiHETrE	""	nM	0.221 ± 0.059	0.202 ± 0.046	0.72	0.47
5,6-DiHETrE	""	nM	0.135 ± 0.046	0.122 ± 0.037	0.76	0.53
17,18-DiHETE	EPA	nM	2.5 ± 1.1	1.79 ± 0.66	1.05	0.10
14,15-DiHETE	""	nM	0.378 ± 0.16	0.247 ± 0.15	1.20	0.057
19,20-DiHDoPE	DHA	nM	0.742 ± 0.32	0.742 ± 0.36	0.14	1.00

All values are mean ± SD. Variable importance in projection scores (VIP) are reported from partial least squares discriminant models of all measured metabolites. P-values are 2-tailed t-tests comparing group means. AA = arachidonic acid; AdA = adrenic acid; αLEA = alpha-linoleic acid; DGLA = dihomo-gamma-linoleic acid; DHA = docosahexaenoic acid; EPA = eicosapentaenonic acid; Fold-C = fold control i.e. Control ÷ HDP; HDP = hemodialysis patient; LA = linoleic acid; OA = oleic acid; PA = palmitic acid; SA = stearic acid.
