# Supplementary material for: Blood Levels of Endocannabinoids, Oxylipins, and Metabolites Are Altered in Hemodialysis Patients
Source: Int J Mol Sci. 2022 Aug 29;23(17):9781. doi: 10.3390/ijms23179781 (PMC9456435; doi:10.3390/ijms23179781)
Supplement: Supplementary file 1 [file ijms-23-09781-s001.zip › New Supplemental Materials Hemo Table S2.pdf]

Supplemental Materials

Table S2. Plasma metabolites measured in healthy controls and hemodialysis patients

Compound name	Hierarchical Cluster	Control Mean ± (SD)	HD Mean ± (SD)	t-test P value	VIP	MANOVA P value
2,3-dihydroxybutanoic acid	2	701 ± (735)	1770 ± (632)	0.0083	1.25	0.0001
2,5-furandicarboxylic acid		207 ± (76.4)	8460 ± (7590)	0.0001	1.24	
2-hydroxyhippuric acid		127 ± (66.9)	1880 ± (2450)	0.04	0.963	
3-(3-hydroxyphenyl)-3-hydroxypropionic acid		231 ± (137)	3690 ± (4630)	0.004	0.975	
3-hydroxy-3-methylglutaric acid		356 ± (85.7)	675 ± (349)	0.0068	1.06	
5-hydroxymethyl-2-furoic acid		199 ± (55.6)	1160 ± (1000)	0.0003	1.15	
cystine		21000 ± (5690)	33700 ± (12100)	0.0062	1.18	
digalacturonic acid		172 ± (62.6)	1890 ± (3330)	0.0006	0.711	
furoylglycine		330 ± (255)	2660 ± (2300)	0.0002	1.19	
glutaric acid		240 ± (21.3)	340 ± (89.2)	0.0028	1.24	
hippuric acid		4020 ± (1400)	148000 ± (127000)	0.0001	1.27	
N-acetylglycine		358 ± (113)	583 ± (304)	0.014	0.844	
orotic acid		173 ± (31.7)	1220 ± (2330)	0.0001	0.625	
paracetamol		410 ± (418)	5470 ± (7900)	0.0033	0.871	
phenylethylamine		788 ± (476)	1100 ± (446)	0.3	0.705	
quinic acid		979 ± (965)	5270 ± (5960)	0.025	0.931	
ribonic acid		400 ± (178)	4170 ± (4460)	0.0004	1.06	
salicylic acid		1650 ± (2000)	2980 ± (5290)	0.8	0.417	
trans-4-hydroxyproline		1860 ± (939)	4010 ± (2870)	0.002	0.933	
1-methyladenosine	4	102 ± (26.5)	555 ± (231)	0.0001	1.63	0.0001
1-methylhydantoin TMS		705 ± (166)	1230 ± (336)	0.0004	1.45	
2-aminoadipic acid		195 ± (51.7)	404 ± (222)	0.0009	1.09	
2-deoxyerythritol		2870 ± (1090)	18100 ± (10400)	0.0001	1.47	
2-deoxyribonic acid		232 ± (95.3)	4490 ± (2220)	0.0001	1.61	
2-deoxytetronic acid		542 ± (134)	2170 ± (1130)	0.0003	1.46	
2-hydroxyglutaric acid		753 ± (137)	1370 ± (578)	0.0027	1.2	
3-hydroxy-3-indoleacetic acid		217 ± (37.9)	1450 ± (491)	0.0001	1.72	
3-methoxytyrosine		300 ± (83.7)	562 ± (145)	0.0004	1.52	
4-hydroxyhippuric acid		160 ± (114)	3640 ± (2270)	0.0001	1.49	
4-hydroxyphenylacetic acid		154 ± (30.1)	716 ± (422)	0.0001	1.39	
5-hydroxy-3-indoleacetic acid		170 ± (16.2)	1360 ± (483)	0.0001	1.72	
5-hydroxyindole-3-acetic acid		109 ± (32.4)	434 ± (273)	0.0021	1.29	
5-hydroxynorvaline		525 ± (211)	731 ± (344)	0.09	0.791	
aconitic acid		240 ± (121)	1110 ± (825)	0.0052	1.24	
adipic acid		617 ± (146)	1570 ± (1180)	0.0015	1.03	

alpha ketoglutaric acid		176 ± (78.2)	370 ± (112)	0.0018	1.44	
arabitol		563 ± (135)	4530 ± (2100)	0.0001	1.61	
beta-alanine		1770 ± (579)	3120 ± (849)	0.0013	1.41	
beta-gentiobiose		308 ± (86.2)	1170 ± (608)	0.0001	1.43	
cellobiose		247 ± (110)	618 ± (256)	0.0004	1.32	
cellobiotol		1620 ± (1060)	2520 ± (2810)	0.3	0.525	

Continuation of Table S2.

Compound name	Hierarchical Cluster	Control Mean ± (SD)	HD Mean ± (SD)	t-test P value	VIP	MANOVA P value
citramalic acid	4	221 ± (187)	915 ± (414)	0.0002	1.49	0.0001
citrulline		994 ± (335)	2110 ± (935)	0.0044	1.35	
creatinine		5430 ± (2830)	58700 ± (38200)	0.0006	1.44	
cysteine		2730 ± (914)	3460 ± (1390)	0.2	0.646	
cysteine-glycine		476 ± (164)	799 ± (500)	0.1	0.881	
deoxytetronic acid		1720 ± (772)	8040 ± (3290)	0.0001	1.6	
erythritol		2410 ± (527)	15200 ± (6160)	0.0007	1.66	
fructose		3760 ± (3450)	8060 ± (7270)	0.1	0.796	
fucose		1020 ± (156)	2540 ± (1690)	0.0005	1.11	
fumaric acid		309 ± (111)	764 ± (383)	0.0004	1.28	
galactinol		594 ± (281)	1600 ± (701)	0.0014	1.42	
galactonic acid		870 ± (149)	5180 ± (3200)	0.0001	1.39	
gluconic acid		832 ± (129)	5840 ± (3460)	0.018	1.45	
gluconic acid lactone		602 ± (114)	1510 ± (469)	0.0001	1.62	
glucuronic acid		2190 ± (1840)	50300 ± (32500)	0.0001	1.46	
glutamic acid		16500 ± (7360)	25500 ± (14800)	0.1	0.794	
glyceric acid		2790 ± (522)	3770 ± (844)	0.0067	1.28	
glycerol-3-galactoside		1320 ± (646)	6850 ± (4030)	0.0004	1.43	
hexuronic acid		1560 ± (302)	4450 ± (2440)	0.0003	1.32	
hypoxanthine		732 ± (310)	1700 ± (558)	0.0005	1.48	
indole-3-acetate		2550 ± (928)	5330 ± (2590)	0.0068	1.25	
indole-3-lactate		1450 ± (574)	4030 ± (2760)	0.0014	1.09	
isocitric acid		1630 ± (295)	3990 ± (1310)	0.0003	1.6	
isothreonic acid		1920 ± (436)	23400 ± (10600)	0.0001	1.64	
kynurenine		361 ± (87.7)	915 ± (327)	0.0001	1.56	
lactobionic acid		298 ± (79.7)	906 ± (593)	0.0013	1.21	
levoglucosan		2030 ± (2150)	26800 ± (30300)	0.0002	1.04	
maltose		5730 ± (3950)	9110 ± (10900)	0.3	0.499	
mannitol		6740 ± (8310)	58100 ± (41900)	0.0009	1.33	
mannonic acid		130 ± (31.2)	394 ± (252)	0.025	1.22	

methylcitrate		103 ± (34.6)	374 ± (134)	0.0001	1.65	
N-acetyl-D-mannosamine		319 ± (67.5)	959 ± (384)	0.0002	1.53	
naproxen		3400 ± (9880)	11900 ± (34300)	0.05	0.463	
ornithine		31200 ± (6940)	56700 ± (17800)	0.0003	1.36	
phenylalanine		31000 ± (5560)	35100 ± (9450)	0.3	0.443	
pseudo uridine		3300 ± (725)	26700 ± (11200)	0.0001	1.66	
pyrazine 2,5-dihydroxy		365 ± (107)	460 ± (115)	0.08	0.757	
rhamnose		1920 ± (834)	4860 ± (3710)	0.013	1.02	
ribose		153 ± (41.8)	861 ± (388)	0.0001	1.59	
saccharic acid		321 ± (113)	4430 ± (2330)	0.0001	1.56	
sorbitol		554 ± (273)	14000 ± (18000)	0.0001	0.972	
sucrose		643 ± (779)	25000 ± (38700)	0.0006	0.841	
threitol		888 ± (216)	10300 ± (4690)	0.0001	1.64	
thymine		247 ± (63.2)	429 ± (145)	0.0093	1.35	

Continuation of Table S2.

Compound name	Hierarchical Cluster	Control Mean ± (SD)	HD Mean ± (SD)	t-test P value	VIP	MANOVA P value
trehalose		1550 ± (857)	2510 ± (2630)	0.4	0.584	
UDP-glucuronic acid	4	1070 ± (1350)	9430 ± (10100)	0.0006	1.05	0.0001
xylitol		528 ± (191)	1100 ± (290)	0.0002	1.51	
2-hydroxybutanoic acid		21600 ± (7030)	13900 ± (4560)	0.016	1.1	
3-phosphoglycerate		529 ± (226)	402 ± (110)	0.3	0.552	
adenosine-5-phosphate		2190 ± (1480)	598 ± (196)	0.0019	1.06	
maltotriose		2790 ± (2790)	784 ± (264)	0.06	0.807	
phosphoethanolamine	8	1920 ± (1360)	1620 ± (433)	0.5	0.255	0.014
pyrophosphate		14600 ± (7550)	7200 ± (1270)	0.012	0.98	
taurine		10700 ± (3910)	3930 ± (1610)	0.0001	1.33	
tocopherol alpha		12200 ± (3930)	9220 ± (1940)	0.06	0.768	
3-aminoisobutyric acid		1690 ± (532)	12100 ± (16600)	0.0005	0.855	
4-hydroxyproline		3310 ± (5610)	10600 ± (15600)	0.5	0.606	
6-deoxyglucitol		1220 ± (234)	2720 ± (1970)	0.0006	0.967	
arabinose		892 ± (108)	5810 ± (8210)	0.0019	0.845	
arachidonic acid		1100 ± (307)	1310 ± (544)	0.5	0.55	
asparagine		4590 ± (1370)	6710 ± (3750)	0.09	0.828	
azelaic acid	3	201 ± (55.8)	260 ± (138)	0.3	0.676	0.025
conduritol betat epoxide		954 ± (414)	2550 ± (1870)	0.031	1.14	
ethanolamine		9780 ± (1770)	13200 ± (6000)	0.3	0.915	
glutamine		107000 ± (42500)	144000 ± (55100)	0.1	0.783	
glycerol-alpha-phosphate		748 ± (203)	806 ± (414)	0.9	0.427	
glycocyamine		342 ± (144)	767 ± (933)	0.4	0.698	

inositol allo-		442 ± (112)	7450 ± (14400)	0.0029	0.877	
lauric acid		6160 ± (1220)	14000 ± (8900)	0.0019	1.07	
lysine		66400 ± (15800)	78200 ± (18100)	0.2	0.754	
methylhexadecanoic acid		2830 ± (928)	3350 ± (1900)	0.6	0.495	
phenylacetic acid		352 ± (111)	465 ± (234)	0.3	0.791	
stearic acid		223000 ± (61300)	226000 ± (60200)	1	0.217	
succinic acid		2020 ± (234)	2370 ± (880)	0.6	0.859	
tartaric acid		869 ± (340)	1650 ± (1690)	0.4	0.75	
2-hydroxyvaleric acid	7	5110 ± (2240)	3900 ± (1300)	0.09	0.507	0.028
2-ketoisocaproic acid		532 ± (130)	397 ± (95.9)	0.02	1.04	
alanine		362000 ± (88000)	220000 ± (42100)	0.0001	1.25	
dodecane		987 ± (224)	718 ± (176)	0.0099	1	
isoleucine		64100 ± (19400)	66600 ± (15400)	0.6	0.306	
lactic acid		199000 ± (87800)	151000 ± (62100)	0.2	0.565	
leucine		119000 ± (38700)	106000 ± (23300)	0.4	0.53	
malic acid		1030 ± (577)	826 ± (201)	0.4	0.347	
proline		146000 ± (61500)	163000 ± (82500)	0.7	0.445	
tyrosine		77300 ± (22400)	63600 ± (11600)	0.1	0.779	
valine		202000 ± (44200)	167000 ± (60600)	0.1	0.874	

Continuation of Table S2.

Compound name	Hierarchical Cluster	Control Mean ± (SD)	HD Mean ± (SD)	t-test P value	VIP	MANOVA P value
1-monopalmitin	1	264 ± (86.9)	204 ± (70.4)	0.1	0.685	0.2
1-monostearin		216 ± (77.7)	169 ± (108)	0.2	0.443	
2-hydroxy-2-methylbutanoic acid		512 ± (386)	693 ± (291)	0.038	0.535	
3-hydroxybutanoic acid		7940 ± (4360)	15900 ± (12700)	0.08	0.737	
4-hydroxybenzoate		1250 ± (3180)	1910 ± (1530)	0.0077	0.376	
4-hydroxymandelic acid		181 ± (43.3)	825 ± (673)	0.0005	1.15	
aminomalonic acid		7510 ± (3430)	6420 ± (1650)	0.5	0.427	
arachidic acid		4170 ± (633)	4480 ± (1410)	0.5	0.129	
benzoic acid		17000 ± (3180)	17700 ± (3750)	0.7	0.208	
citric acid		61900 ± (17000)	63800 ± (17600)	0.8	0.263	
cyclohexylamine		955 ± (263)	1400 ± (507)	0.016	0.993	
cytidine-5'-diphosphate		511 ± (229)	666 ± (240)	0.1	0.683	
dodecanol		483 ± (161)	462 ± (81)	1	0.261	
gamma-tocopherol		2570 ± (1620)	3380 ± (1340)	0.2	0.475	
glucose		485000 ± (72500)	440000 ± (62800)	0.2	0.697	
glycine		350000 ± (119000)	385000 ± (141000)	0.5	0.492	
glycolic acid		2270 ± (564)	2400 ± (530)	0.6	0.458	

homocystine		314 ± (70.5)	357 ± (142)	0.6	0.514	
homovanillic + 4-hydroxymandelic acid		184 ± (52.8)	778 ± (550)	0.0002	1.24	
mevalonic acid		374 ± (184)	560 ± (137)	0.024	0.994	
palatinitol		137 ± (93.1)	112 ± (52.7)	0.6	0.433	
phosphoric acid		116000 ± (39800)	88600 ± (69600)	0.1	0.469	
threonic acid		13900 ± (7270)	16300 ± (6010)	0.5	0.408	
threonine		57000 ± (12200)	52600 ± (21900)	0.7	0.356	
tocopherol beta		398 ± (110)	701 ± (522)	0.012	0.758	
uridine		419 ± (117)	423 ± (160)	0.9	0.179	
vanillic acid		129 ± (49.6)	588 ± (733)	0.0005	0.863	
1,5-anhydroglucitol	5	64200 ± (32500)	27200 ± (18100)	0.0018	1.14	0.2
capric acid		2600 ± (544)	2240 ± (536)	0.1	0.714	
cholesterol		400000 ± (102000)	332000 ± (58000)	0.08	0.657	
glycerol		56300 ± (21100)	68400 ± (49500)	0.7	0.408	
histidine		20500 ± (6700)	20800 ± (5540)	0.9	0.227	
inulotriose		610 ± (512)	439 ± (177)	0.7	0.323	
lathosterol		316 ± (141)	200 ± (79.3)	0.02	0.846	
linoleic acid		3150 ± (1840)	3380 ± (1560)	0.6	0.171	
maleimide		1160 ± (325)	946 ± (183)	0.1	0.599	
methanolphosphate		2110 ± (701)	1340 ± (208)	0.0036	1.03	
methionine sulfoxide		7710 ± (1870)	7780 ± (2870)	1	0.272	
myristic acid		3180 ± (991)	3220 ± (1060)	0.6	0.235	
N-acetylglutamate		714 ± (411)	676 ± (274)	0.8	0.0565	
octadecanol		2260 ± (5350)	2710 ± (6360)	0.7	0.124	
oleic acid		9920 ± (5770)	11300 ± (8090)	0.5	0.276	
oxoproline		110000 ± (26200)	115000 ± (29500)	0.7	0.525	
palmitic acid		50500 ± (12600)	53800 ± (14800)	0.5	0.417	
palmitoleic acid		5260 ± (4220)	4430 ± (4670)	0.7	0.231	

Continuation of Table S2.

Compound name	Hierarchical Cluster	Control Mean ± (95%CI)	HD Mean ± (95%CI)	t-test P value	VIP	MANOVA P value
pelargonic acid	5	11700 ± (2000)	10800 ± (1800)	0.3	0.384	0.2
pimelic acid		1150 ± (394)	868 ± (426)	0.1	0.572	
propane-1,3-diol		1970 ± (637)	1220 ± (391)	0.0066	1.04	
tryptophan		53000 ± (19800)	30300 ± (9080)	0.0038	1.1	
uric acid		45500 ± (22400)	47200 ± (16600)	0.9	0.135	
2,3,5-trihydroxypyrazine	6	156 ± (110)	159 ± (75.1)	0.6	0.172	0.7
5-methoxytryptamine		1890 ± (2180)	666 ± (438)	0.3	0.635	
allantoic acid		510 ± (165)	658 ± (167)	0.08	0.697	
aspartic acid		3840 ± (1660)	2180 ± (717)	0.1	0.965	
hydroxycarbamate		1310 ± (560)	1740 ± (412)	0.06	0.732	
hydroxylamine		4160 ± (1600)	5580 ± (1520)	0.07	0.762	
methionine		2230 ± (1440)	1190 ± (962)	0.018	0.961	
nicotinic acid		25400 ± (12000)	28100 ± (18000)	0.7	0.193	
N-methylalanine		13300 ± (5880)	10100 ± (4330)	0.3	0.698	
oxalic acid		766000 ± (337000)	830000 ± (369000)	0.7	0.315	
parabanic acid		1810 ± (913)	2280 ± (782)	0.3	0.439	
serine		37600 ± (10900)	29500 ± (6340)	0.1	0.818	
shikimic acid		1130 ± (1120)	917 ± (515)	1	0.34	
trihydroxypyrazine		334 ± (126)	317 ± (204)	0.6	0.704	
urea		480000 ± (172000)	232000 ± (184000)	0.0077	1.18	
